# The Influence of Hepatitis C Virus Infection on ORAL Health-Related Quality of Life in Patients with Oral Lichen Planus

**DOI:** 10.3390/ijerph18179382

**Published:** 2021-09-06

**Authors:** Doina Iulia Rotaru, Radu Marcel Chisnoiu, Andreea Iuliana Kui, Sorana D. Bolboacă, Andrea Maria Chisnoiu

**Affiliations:** 1 Department of Odontology, Endodontics and Oral Pathology, “Iuliu Hațieganu” University of Medicine and Pharmacy, 400001 Cluj-Napoca, Romania; doina.rotaru@umfcluj.ro; 2 Department of Prosthodontics, “Iuliu Hațieganu” University of Medicine and Pharmacy, 400006 Cluj-Napoca, Romania; maria.chisnoiu@umfcluj.ro; 3 Department of Medical Informatics and Biostatistics, “Iuliu Hațieganu” University of Medicine and Pharmacy, 400349 Cluj-Napoca, Romania; sbolboaca@umfcluj.ro

**Keywords:** oral lichen planus, hepatitis C virus, quality of life (QoL), OHIP-14 questionnaire

## Abstract

Background: oral lichen planus (OLP) is a mucocutaneous disease that affects about 4% of the global population. Hepatitis C virus (HCV) was linked to lichen planus. The current study aimed to assess the impact of OLP associated or not with HCV infection on the oral health-related quality of life (OHRQoL). Methods: The study included patients diagnosed with OLP who filled in the Romanian version of OHIP-14 questionnaire at their first appointment and 6 months later, after the OLP treatment. A control group of OLP-free subjects similar as age and sex was also included in the study. *Results*: 68 patients with OLP and 46 controls were included in the study. The OHIP scores are significantly higher on OLP group than controls (*p*-value < 0.0001) and significantly reduced at 6-month follow-up (*p*-values: < 0.0001 pre- vs. post-treatment in OLP group). Patients with and without HCV associated with OLP demonstrated a similar oral quality of life (*p*-values > 0.05). *Conclusions*: the OHRQoL for patients suffering from OLP is compromised but is improved after treatment. The HCV associated with OLP did not influence the overall OHRQoL, but the patients who associate HCV reported more frequently aching in the mouth and discomfort eating food at six-month follow-up.

## 1. Introduction

Oral lichen planus (OLP) is a mucocutaneous disease that affects about 4% of the global population. It is more common in women, with a prevalence of 2:1 (women:men), and primarily affects the adults [1,2]. The etiology of this disease is still uncertain, but the initial triggering factors are an autoimmune response to local antigens, inducers of cell-mediated hypersensitivity, microorganisms, and even stress [3].

The patients suffering from OLP may have no symptomatology, or they may have a sensation of mucosal roughness to burning sensation and pain that influence their daily activities and physiological functions like eating, swallowing, or speaking. The natural course of OLP involves a succession of active phases of the disease and remission periods. As such, there are delays in addressing the medical services, which hinders an early diagnosis. Moreover, because the current treatments only reduce the clinical manifestations of the disease and do not completely heal, the patient’s motivation in addressing the physician is reduced. Consequently, the subsequent surveillances are difficult [4]. Usual OLP treatment relies on corticosteroids administrated topically or systemically [5]. A treatment using platelet-rich fibrine injections proved to reduce the extension of OLP and its symptomatology [6].

According to Andreasen’s clinical classification, six forms of OLP are known: reticular, erosive-ulcerative, papular, atrophic, plaque, and bullous [7]. Reticular form is considered to be a “mild” form, while the erosive-ulcerative is a “severe” one and can be a life-threatening affection, preventing the patients from eating and drinking due to the painful symptomatology.

Hepatitis C virus (HCV) was linked to lichen planus, especially with the involvement of the oral cavity [8]. Generally, HCV is associated with a broad spectrum of biological and clinical extrahepatic manifestations [9]. About 20% of patients suffering from C hepatitis may develop lichen planus [10]. A few reports show the OLP symptoms decrease the quality of life for the OLP patients with HCV-related liver disease [11]. Regarding the painful OLP, it is obvious that the oral cavity of the HCV-positive patients is more likely to become less healthy than those of the HCV-negative ones [12,13]; that is why a hepatologist must collaborate with an oral pathologist or a dentist to educate the patients regarding oral hygiene so the HCV-positive patient’s quality of life does not decrease.

To assess the physical, social, and psychological consequences of oral health and the impact of oral health status on quality of life, different patient-centered oral health status measurements were developed. In 1994, Slade and Spencer developed and validated the Oral Health Impact Profile (OHIP) [14]. The Oral Health Impact Profile (OHIP) is a questionnaire consisting of 49 questions (OHIP-49) grouped into 7 dimensions: functional limitation, physical pain, psychological discomfort, physical disability, psychological disability, social disability, and handicap. The disadvantage of this questionnaire was its length, being a time-consuming element for both the patient and the clinician. Slade developed in 1997 a shorter version consisting of just 14 questions (OHIP-14), where 2 items are used to measure the patient’s responses for each of the 7 dimensions [15]. The Romanian version of OHIP-14 was reported by Murariu et al. and was validated with an adult population in Iași [16].

The present study aimed to assess the impact of oral lichen planus lesions on the oral health-related quality of life (OHRQoL) pre- and post-OLP treatment in the context of hepatitis C infection.

## 2. Materials and Methods

### 2.1. Design and Settings

The study included patients who addressed the Department of Odontology, Endodontics and Oral Pathology, Faculty of Dental Medicine, “Iuliu Hațieganu” University of Medicine and Pharmacy, Cluj-Napoca, Romania, between January 2016–December 2020, for dental examination and treatment.

The inclusion criteria in the study were:

- Age older than 18 years
- The diagnosis of OLP based on clinical and histopathological criteria established by World Health Organization and modified by van der Meij and van der Waal [17]
- Expressing the informed consent to participate in the study

The patients with psychoactive medication or acute, painful conditions were excluded.

The controls were selected from the patients who attended the same institution in the same period; patients who came for a specialist examination but did not present any oral mucosa lesions, and those who were similar in regards of age and sex with the cases.

Demographic data (e.g., gender, age, living location) and oral mucosal lesions (e.g., type of lesions—reticular/atrophic/erosive, location, associated pain) were recorded based on the clinical examination. The association or absence of HCV infection was also recorded.

The patients were asked to fill in the Romanian version of the OHIP-14 questionnaire to assess subjective OHRQoL. This questionnaire contained 14 questions grouped in 7 domains—functional limitation, physical pain, psychological discomfort, physical disability, psychological disability, social disability, and handicap. The possible answers are given on a five-point Likert scale: 0 = never, 1 = hardly ever, 2 = occasionally, 3 = fairly often, 4 = often. The OHIP-14 overall score takes value from zero, indicating better OHRQol, to 56, indicating a worse OHRQol.

The clinical examination and the questionnaire were repeated after six months for each patient. The baseline and follow-up clinical examination were completed by the same physician.

The study protocol was reviewed and approved by the Ethics Committee of the “Iuliu Hațieganu” University of Medicine and Pharmacy, Cluj-Napoca, Romania (approval no. 160/2.04.2018).

### 2.2. Statistical Analysis

Statistical analysis was performed using the Statistica program (StatSoft USA, v. 8). Age variable was tested for normality with the Shapiro–Wilk test at a significance level of 5% and results were reported as median (Q1 to Q3) where Q1 is the 25th percentile, and Q3 is the 75th percentile. Qualitative data were reported as numbers and percentages. Nonparametric tests for independent (between OLP subgroups or between OLP and controls; Mann–Whitney Test) or respectively dependent samples (pre- and post-treatment; Wilcoxon Matched Pairs Test) were applied to compare groups. The *p*-values smaller than 0.05 were considered statistically significant.

## 3. Results

**Figure 1:**
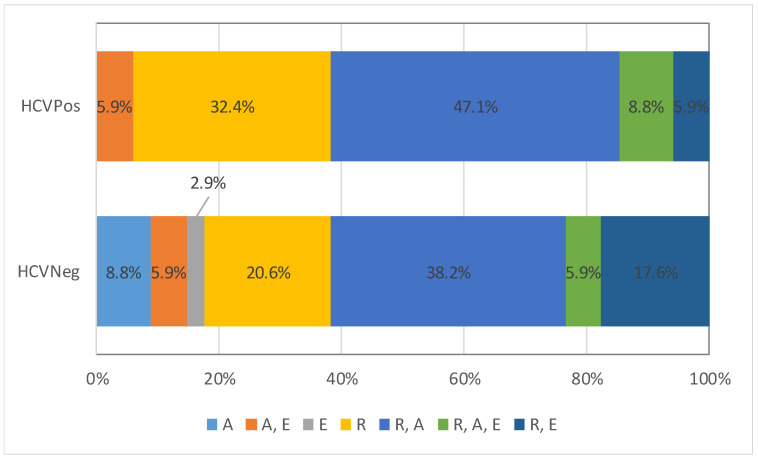
Type of lesions in OLP group: differences between those with and without HCV (A = Atrophic, E = Erosive, R = Reticular).

Sixty-eight subjects with OLP, aged from 30 to 77 years, and 46 controls, aged from 30 to 72 years, were included in the analysis. No significant differences regarding gender, age, or environment were observed between the group with OLP and the controls (Table 1). Among the group with OLP, no significant differences were identified when the HCV-positive (HCVPos) patients were compared with the HCV-negative (HCVNeg) patients (Table 1). Regardless of the HCV status, most frequently, the patients with OLP had mixed reticular and atrophic lesions (Figure 1).

No changes were observed regarding the type of lesions at follow-up among those with OLP, but the OHIP-14 scores were significantly reduced after treatment (Table 2), with comparable values in the control group. No significant differences in the OHIP score both pre- and post-treatment were observed among women as compared to that of men (Mann–Whitney Test—pre-: *p*-value = 0.1272; post-treatment: *p*-value = 0.3460).

The scores in pretreatment among those with OLP in most of the cases remained the same or reduced after treatment, with few exceptions (Table 3). The post-treatment scores were higher than that of the pretreatment scores for one or two patients in most cases for HCVPos patients (Table 3). The post-treatment scores were significantly smaller than that of the pre-treatment scores, with two exceptions represented by difficulty relaxing and embarrassment (Table 3).

**Figure 2:**
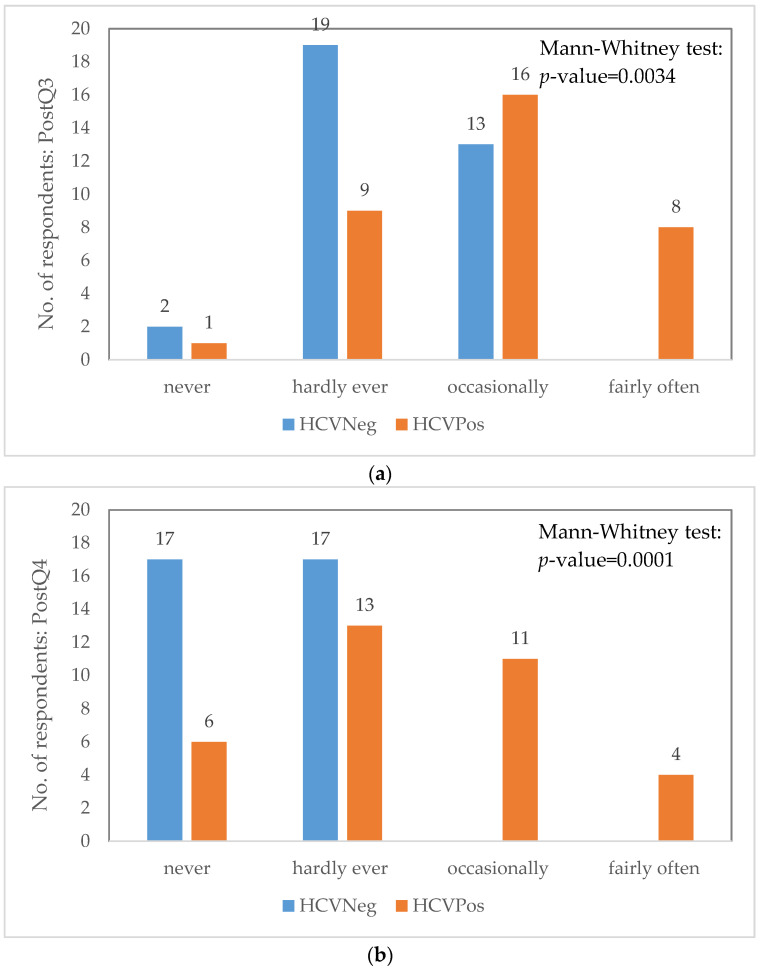
Differences in trends among OLP patients (Post = Post-treatment score; Q3 = Aching in mouth; Q4 = Discomfort eating food).

The comparison within the OLP among those HCVPos and HCVNeg identified several significant differences regarding the scores for individual questions of the OHIP-14 as presented in Figure 2.

## 4. Discussion

The quality of life of patients with OLP proved significantly lower than that of controls, significantly improved after treatment, and became similar with the control group at six months follow-up. The OLP lesion type and the quality of life are similar among HCV-positive and HCV-negative patients.

The association of OLP with chronic liver disease was suggested since 1980. Since then, much research was conducted supporting this connection. The prevalence of chronic hepatitis among the OLP patients is reportedly between 0.5–35%, depending on the different geographic areas [18,19].

Regarding the patients with HCV-associated liver disease, Nagao et al. reported that zinc levels and the sensitivity to tastes are decreased. Moreover, some of the patients were unaware of their taste disorder [20]. The current study results did not reveal any taste modification pre- and post-OLP treatment or between HCV positive and negative groups (Table 3).

Since OLP is a dynamic disease with different clinical forms over time, usually evolving from mild-to-severe forms, a continuous follow-up is necessary [21,22]. So, the education level might affect the awareness for the importance of periodic and continuous medical check-ups and, consequently, the psychological well-being and socioeconomic status among OLP patients. The result of our study showed a significant improvement of OHRQoL at six-month follow-up (Table 2).

The OHIP is a questionnaire designed to measure self-reported discomfort, dysfunction, and disability due to oral conditions. As a conceptual base, it has an oral health model outlined by Locker et al. [23]. It was proven to be sensitive to changes and reliable, and to exhibit suitable cross-cultural consistency. Unfortunately, routinely, the patient-based outcomes are not incorporated into the clinical decision-making process. The evidence-based medicine should mean that the patients’ perceptions of quality of life might be included in this process. This would increase the doctors’ awareness of how the patients’ daily lives are affected by the disease. When only clinical measures are taken into account, the impact of oral health on patients’ quality of life does not have an accurate representation and, if the treatment targets only the physiological factors, it may not be as effective as when considering all aspects of health [24]. Any treatment should consider not only extending the lifetime, but, also, improving the patients’ appearance, interpersonal relationships, and individual positive self-image [25].

OHIP-14 is considered reliable and valid in OLP patients from different populations, being an instrument with good psychometric properties [26,27]. It is shorter than the original OHIP-49, and it can be easily used in clinical practice. Even if it is not a disease-specific instrument, it is beneficial in detecting the evolution and the changes that occur over time. It is sensitive to dental, periodontal, temporomandibular joint, or any other oral mucosa disorder [26,28,29,30,31,32].

The present study results illustrated that OLP has a negative impact on OHRQoL, the patients having a poor quality of life compared to the healthy subjects (Table 2 and Table 3). This is mainly due to burning sensation, pain and, in addition, frustration induced by the difficulties faced by the clinicians in providing a satisfactory treatment because of the unclear etiology, variable symptoms and clinical signs of this affection [33,34]. OHRQoL is also influenced by different diagnostic criteria, the clinical form of OLP, and the heterogeneity of OLP population.

The study results need to be compared to that of OHIP-14 reference scores. John et al. reported an average OHIP-14 score of 4.09 for the general population in Germany [34]. In our study, half of the subjects in the control group had an OHIP-14 score less than or equal to 9.5 (Table 2), while the post-OLP treatment scores remained slightly higher compared to that of controls (Table 2). The OHIP-14 score reported in our study is higher compared to that of the one reported by John et al. [34] and could be explained by the fact that our controls were not selected from the general population.

In the present study, the OLP patients achieved a pretreatment median score of 18.5, including the HCV-infected ones (Table 2) that show a significant reduction in the quality of life of these patients. Our result is similar to scientific publications that place the average OHIP-14 score of OLP patients between 9.42 and 21.7 [4]. The variation of OHIP-14 score of OLP patients is attributed to the methodology, especially concerning the differences in study design and the population [4]. According to the current study, no significant differences were noticed between HCVPos and HCVNeg patients (Table 2). To the best of our knowledge, no other study to compare the OHRQol between HCVPos and HCVNeg OLP patients was reported in the scientific literature.

Regarding the post-treatment scores, our results showed a significant improvement of OHRQoL for all OLP patients (Table 2). This is also in accordance with other studies that concluded an improvement in quality of life regardless of the therapy form [35,36].

The results of the present study are in accordance with the ones obtained by McGrath et al. [2], who described the sensitivity of OHIP-14 for the assessment of clinical effects in OLP treatment and noticed decreased pain following the treatment. Referring to the ratio between affected women and men, no significant difference between the two categories was found in this study, which contradicts the observations of Karbach et al. [4], who concluded that women are more affected. However, Aksoy et al. [37], pointed out that this difference can be attributed to the proportion of females included in the studies. The patients included in the present study were more affected by the presence of pain or limitation of oral function (Table 3, Figure 2), similar to the previously reported results [4,37].

Considering the form of OLP, in the current study, the reticular form is the predominant one (Table 1). This is known as a “mild” form, while the other forms are categorized as “severe” forms [38]. The reports regarding OHRQoL related to the form of OLP are heterogenous. Parlatescu et al. reported an OHIP-14 score of 11.77 for the patients with reticular OLP and 15.68 for erosive, atrophic, ulcerative, and bullous forms [39]. Aksoy et al. reported a score of 8 for the patients with reticular OLP and a score of 11 for the nonreticular OLP patients and concluded that the quality of life is decreased most in the presence of pain or oral functions impairment [38]. Vilar–Villanueva et al. found higher scores for ulcerative and atrophic forms when compared to reticular OLP [40], while Karbach et al. reported similar findings, but with lower scores [4]. Hagarty et al. [41] pointed out that the impact of erosive OLP lesions was significant, and most patients suffered from psychological and social consequences. The severe forms cause pain and lead to restrictions in eating, swallowing, speaking, and oral care, while anxiety and depression are common cofactors in OLP [42]. Moreover, the potential risk of malignant transformation associated with OLP, has an extra negative influence on patients’ quality of life. Psychological factors play an important role in OLP progression and the patients are more stressed, anxious, and prone to depression [43].

In the literature, a limited number of studies assessed the effects of OLP on health-related quality of life. Zou et al. [44], using the Chinese version of OHIP-14, concluded that the OLP patients have a lower quality of life, but they did not report any data regarding the type of OLP. Similar results are also reported by Lopez–Jornet et al. [45], with low-quality of life among OLP patients comparing with the control group, but without counting the type of lesions.

Overall, in the current study, the OHRQoL for HCVPos patients varied similarly with that for HCVNeg patients, with few exceptions but without statistical significance (Table 3). What was different and statistically validated was the post-treatment status regarding the aching in the mouth and, consequently, discomfort in eating for the HCVPos patients, which confirms the results reported by Coates et al. [12] and Henderson et al. [13].

The current study is the first to assess the quality of life for the patients suffering from OLP in the northwest of Romania using the validated Romanian version of OHIP-14 questionnaire. Moreover, based on our access to the scientific literature, our study seems to be the first study that assesses the OHRQoL separately for the HCVPos patients in Romania.

Not considering possible confounding factors (e.g., comorbid systemic disease, presence of dentures, smoking, alcohol consumption, socioeconomic status, education, etc.) [46] with an impact on OHRQoL could be seen as the main limitation of our study. The self-reported data are known to be affected by social desirability (cultural norms declaration instead of reality), acquiescence (tendency to be agreed), leniency or harshness (systematic positive or negative answers), and recall bias [47]. Furthermore, our patients received recommendations regarding the effects of smoking and alcohol consumption to avoid OLP exacerbation, so asking for these habits was not considered. Methodological techniques, such as longitudinal study or combination of an interview with self-reported survey [47], or statistical techniques to identify response bias, such as stochastic frontier estimation [48] or to confounding adjustment [49,50], were proposed in the scientific literature. All such methods increase the costs and duration of the study and do not fit pilot or feasibility studies. OLP etiology is various and frequently difficult to establish with high accuracy. Larger samples are required to evaluate all possible individual confounding factors as stratified based on etiology. Furthermore, an evaluation by a populational study of OHIP-14 score for the general population would also be beneficial.

The use of oral health-related quality of life measurements is important for screening as it was demonstrated to be a relevant tool and should be included in patient’s therapy. While the clinically visible parameters are insufficient to evaluate a patient condition and to conduct a therapy, the patient’s whole well-being should be considered. Considering the limitations of this study we still believe that our results support the use of quality-of-life measurements in oral medicine.

## 5. Conclusions

Our study demonstrated significantly higher OHIP-14 scores among patients with OLP compared to that of controls, scores that significantly decreased after treatment, with slighted but not significantly higher values at the six-month follow-up as compared to that of controls. Excepting aching in mouth and discomfort eating food, which are reported more frequently by OLP patients with hepatitis C virus (HCV) infection, OLP-HCV association had no overall effect on OHRQoL.

## Figures and Tables

**Table 1:** Characteristics of investigated groups.

	Oral Lichen Planus (*n* = 68)				Control (*n* = 46)	
	All	HCVPos (*n* = 34)	HCVNeg (*n* = 34)	*p*-Value ^a^	Value	*p*-Value ^b^
Women, *n* (%)	39 (57)	21 (62)	18 (53)	0.462	26 (57%)	0.9299
Age (years)				0.4883		0.2788
median	56	54	57.5		55	
(Q1 to Q3)	(42.75 to 66)	(41.25 to 65)	(43.25 to 67.75)		(41 to 61.75)	
Rural zone, *n* (%)	30 (44)	16 (47)	14 (41)	0.6252	21 (46)	0.8716
Cutaneous lesions = yes, *n* (%)	40 (59)	23 (68)	17 (50)	0.1393	0	n.a.
Lesion type						
Reticular	60 (88)	32 (94)	28 (82)	0.2585	0	n.a.
Atrophic	41 (60)	21 (62)	20 (59)	0.8043	0	n.a.
Erosion	18 (26)	7 (21)	11 (32)	0.2716	0	n.a.

^a^ comparison between HCVPos group with HCVNeg group; ^b^ as compared to that of OLP.

**Table 2:** Oral health-related quality of life comparisons: pre- and post-treatment, and OLP vs. control.

	Oral Lichen Planus				Control	
	All	HCVPos	HCVNeg	*p*-Value **	Value	*p*-Value **
OHIP-14				0.3295		<0.0001
Pre-	18.5 (16 to 21)	18 (16.3 to 19.8)	19 (16.3 to 21)	0.1832	9.5 (0 to 14.8)	0.1479
Post-	11 (9 to 14)	11.5 (10 to 14)	10 (8.3 to 14)			
*p*-value *	<0.0001	<0.0001	<0.0001			

Pre- and Post OHIP-14 scores are expressed as median (Q1 to Q3), where Q is value of the quartile; * Wilcoxon test; ** Mann–Whitney Test.

**Table 3:** Differences between pre- and post-treatment OHIP scores in OLP group.

OHIP	Difference between Pre- and Post-Treatment Score					*p*-Value *
	−1	0	1	2	3	
1: Trouble pronouncing words						
All		56 (82.4)	12 (17.6)			0.0022
HCVPos		28 (82.4)	6 (17.6)			0.0277
HCVNeg		28 (82.4)	6 (17.6)			0.0277
2: Worsened taste						
All		52 (76.5)	15 (22.1)	1 (1.5)		0.0004
HCVPos		25 (73.5)	8 (23.5)	1 (2.9)		0.0077
HCVNeg		27 (79.4)	7 (20.6)			0.018
3: Aching in mouth						
All	1 (1.5)	10 (14.7)	35 (51.5)	18 (26.5)	4 (5.9)	<0.0001
HCVPos	1 (2.9)	6 (17.6)	24 (70.6)	3 (8.8)		<0.0001
HCVNeg		4 (11.8)	11 (32.4)	15 (44.1)	4 (11.8)	<0.0001
4: Discomfort eating food						
All		10 (14.7)	37 (54.4)	16 (23.5)	5 (7.4)	<0.0001
HCVPos		9 (26.5)	25 (73.5)			<0.0001
HCVNeg		1 (2.9)	12 (35.3)	16 (47.1)	5 (14.7)	<0.0001
5: Feeling self-conscious						
All	1 (1.5)	24 (35.3)	32 (47.1)	10 (14.7)	1 (1.5)	<0.0001
HCVPos	1 (2.9)	12 (35.3)	17 (50.0)	4 (11.8)		0.0001
HCVNeg		12 (35.3)	15 (44.1)	6 (17.6)	1 (2.9)	<0.0001
6: Feeling tense						
All		23 (33.8)	34 (50.0)	10 (14.7)	1 (1.5)	<0.0001
HCVPos		11 (32.4)	17 (50.0)	6 (17.6)		<0.0001
HCVNeg		12 (35.3)	17 (50.0)	4 (11.8)	1 (2.9)	<0.0001
7: Poor diet						
All	2 (2.9)	50 (73.5)	14 (20.6)	2 (2.9)		0.0028
HCVPos	1 (2.9)	23 (67.6)	9 (26.5)	1 (2.9)		0.0145
HCVNeg	1 (2.9)	27 (79.4)	5 (14.7)	1 (2.9)		0.0759
8: Interrupted meals						
All	1 (1.5)	46 (67.6)	21 (30.9)			0.0002
HCVPos	1 (2.9)	23 (67.6)	10 (29.4)			0.0164
HCVNeg		23 (67.6)	11 (32.4)			0.0033
9: Difficulty relaxing						
All	2 (2.9)	61 (89.7)	5 (7.4)			0.3105
HCVPos	2 (5.9)	28 (82.4)	4 (11.8)			
HCVNeg		33 (97.1)	1 (2.9)			
10: Embarrassment						
All	1 (1.5)	65 (95.6)	2 (2.9)			0.593
HCVPos	1 (2.9)	31 (91.2)	2 (5.9)			
HCVNeg		34 (100)				
11: Irritability						
All	1 (1.5)	46 (67.6)	21 (30.9)			0.0002
HCVPos		22 (64.7)	12 (35.3)			0.0022
HCVNeg	1 (2.9)	24 (70.6)	9 (26.5)			0.0249
12: Difficulty in doing usual jobs						
All	1 (1.5)	42 (61.8)	24 (36.8)			<0.0001
HCVPos	1 (2.9)	21 (61.8)	12 (35.3)			0.0071
HCVNeg		21 (61.8)	13 (38.2)			0.0015
13: Life less satisfying						
All	1 (1.5)	32 (47.1)	29 (42.6)	5 (7.4)	1 (1.5)	<0.0001
HCVPos	1 (2.9)	16 (47.1)	15 (44.1)	2 (5.9)		0.0008
HCVNeg		16 (47.1)	14 (41.2)	3 (8.8)	1 (2.9)	0.0002
14: Inability to function						
All		28 (41.2)	34 (50.0)	6 (8.8)		<0.0001
HCVPos		16 (47.1)	15 (44.1)	3 (8.8)		0.0002
HCVNeg		12 (35.3)	19 (55.9)	3 (8.8)		<0.0001

* Comparison of pre- and post-treatment scores: Wilcoxon Matched Pairs Test.

## Data Availability

No new data were created or analyzed in this study. Data sharing is not applicable to this article.
